# Beliefs, fear and awareness of women about breast cancer: Effects on mammography screening practices

**DOI:** 10.1002/nop2.696

**Published:** 2020-11-20

**Authors:** Lida Emami, Akram Ghahramanian, Azad Rahmani, Ahmad Mirza Aghazadeh, Tonia C. Onyeka, Amirreza Nabighadim

**Affiliations:** ^1^ Medical Surgical Department, Nursing and Midwifery Faculty Islamic Azad University Bonab Branch Bonab Iran; ^2^ Medical and Surgical Department, Nursing and Midwifery Faculty, Hematology and Oncology Research Center Tabriz University of Medical Sciences Tabriz Iran; ^3^ Medical Surgical Department, Nursing and Midwifery Faculty Tabriz University of Medical Sciences Tabriz Iran; ^4^ Department of Basic sciences Paramedical Faculty Tabriz University of Medical Sciences Tabriz Iran; ^5^ Department of Anaesthesia/Pain & Palliative Care Unit Multidisciplinary Oncology Centre College of Medicine University of Nigeria Ituku‐Ozalla Campus Enugu Nigeria; ^6^ Department of Urology Uro‐Oncology Research Center Tehran University of Medical Sciences Tehran Iran

**Keywords:** beliefs, breast cancer, fatalism, Iran, mammography screening practice, Muslim

## Abstract

**Aim:**

This study sought to investigate the beliefs, fear and awareness about breast cancer and mammography screening practices of women in Iran.

**Methods:**

This descriptive‐correlational study was conducted at Tabriz, East Azerbaijan Province, northwest of Iran from February–July 2017. One hundred and fifty‐two women aged 40 years and older, who were referred to 12 health centres for health services were selected via clustering sampling. Associations between variables and mammography screening practices were examined using bivariate and multivariate logistic regression analyses. Participants who had a mammogram within the last 24 months were compared with those who had none. Sociodemographic questionnaire, Champion's Breast Cancer Fear Scale, Champion's Health Belief Model Scale for Mammography Screening, Breast Cancer Awareness Scale and Powe Fatalism Inventory were the tools used for data gathering.

**Results:**

Just 38.2% of women reported having a mammogram within the last 24 months. Self‐efficacy (OR = 5.36, B = 1.68, *p* < .001), susceptibility (OR = 2.83, B = 1.04, *p* < .001), motivation (OR = 2.11, B = 0.75, *p* = .024) and lower perceived barriers (OR = 0.25, B = −1.37, *p* < .001) were associated with being screened. Neither fatalistic belief nor awareness towards breast cancer was significant.

## 1 INTRODUCTION

For women, of all the most commonly diagnosed cancers (breast, lung and bronchus and colorectal), breast cancer alone is expected to account for 30% of all new cancer diagnoses (Siegel et al., 2017). According to the report of the Iranian National Cancer Registry, 55.58% and 44.41% of cancer cases occurred in the Iranian men and women, respectively, with breast, colorectal and stomach cancer increasing annually by 13.8%, 1.3% and 11.6%, respectively (Rafiemanesh et al., 2016). In addition, 70% of the Iranian women with breast cancer present in an advanced stage (Khodayarian et al., 2016), thereby increasing the national oncologic burden. The burden of breast cancer could be substantially reduced through implementation of screening programmes. In 2012, the estimated overall cancer death rates in general were higher among women in low‐ and middle‐income countries (LMICs) than their counterparts in high‐income countries (HICs), largely due to inadequate access to early detection and treatment (Torre et al., 2017). In Iran, breast cancer presents a different pattern from that in advanced countries and develops at least a decade earlier than for women in those countries (Farhood et al., 2018; Sadjadi et al., 2009). In Iranian women, breast cancer is commonly diagnosed at higher grades with poor prognosis (Babu et al., 2011; Montazeri et al., 2008). Therefore, early screening for breast cancer can be a useful approach to reduce mortality in high‐risk populations (Farhood et al., 2018). The mortality from breast cancer is known to have an inverse relationship with mammography (Herrmann et al., 2018; Morrell et al., 2017). The United States Preventive Services Task Force (USPSTF, 2016) found adequate evidence that mammography screening reduces breast cancer mortality in women aged 40–74 years. The USPSTF recommends biennial screening mammography for women aged 50–74 years. While screening mammography in women aged 40 to 49 years may reduce the risk for breast cancer death, the number of deaths averted is smaller than that in older women and the decision to start screening mammography in women prior to age 50 years should be an individual one. Women who place a higher value on the potential benefit (for example women with a parent, sibling or child with breast cancer) than the potential harms may choose to begin biennial screening between the ages of 40–49 years. However, mortality rates are still high because access to mammography in many LMICs is limited to the large cities (Tavafian et al., 2009). Even in the large cities like Tehran where mammography is available, mammography rates remain low, even among female academics who are also physicians (Saadat et al., 2017).

### 1.1 Literature review

Some studies conducted among women in Southern and Northern Iran have reported rates of mammography practised at least once in their lifetime, to be between 1.3% (Heidari et al., 2008)–21.7% (Kardan‐Souraki et al., 2019) respectively. In one systematic review, it was revealed that many factors reduce the tendency of women to perform mammography and these barriers permeate all levels of society (Sarma, 2015). Some researchers have suggested that these barriers be taken into consideration to understand screening behaviours and promote screening efforts (Akhigbe & Akhigbe, 2012). Some of these factors such as awareness, attitude, cultural values, fear, belief in fate, self‐efficacy and psychosocial factors influence screening behaviour. The findings of a study highlight the importance of understanding perceived susceptibility to cancer, fatalistic beliefs about cancer and information seeking with regard to screening behaviours (Valera et al., 2018). A study demonstrated that even when appropriate medical care is accessible, screening levels among people who have fatalistic beliefs remain low (Peek et al., 2008). In another study, fear and belief in fate were identified as the barriers to breast cancer screening in the African‐American women (Talbert, 2008). A study in Iran (Mokhtari et al., 2011) showed that being a healthcare provider did not influence the decision of women to perform mammograms regularly and the same study reported low mammography rates among the women from fear of detection of a breast lump and perceived fear of physical pain of the mammography procedure despite a display of high knowledge.

In another instance, the Iranian women sampled with no experience of mammography, had little to no knowledge of the BC, blamed the development of the latter on mammography and refused the procedure on the grounds of loss of sexuality, loss of spouse, lack of financial and social support as well as the loss of femininity (Khazaee‐pool et al., 2014). Also, the study by Saadat et al. (2017) indicates that the mammography screening rates among female Iranian doctors are lower than found among their counterparts in other developing countries.

Perceived risk due to the cancer is affected by individuals’ beliefs about cancer and perceived susceptibility to cancer. How individuals perceive their cancer risk is important to understanding their preventive and information‐seeking behaviours (Wigfall & Friedman, 2016). A systematic review (Badakhsh et al., 2018) reported the attitude and practice of the Iranian woman on breast cancer screening to be inappropriate with mammography rates described to be at between 9.1% (Mahoori et al., 2003)–24% (Iurigh et al., 2016). There are instances where Muslim women are refused participation in screening because of cultural and religious beliefs, despite their knowledge on the role of mammograms in early diagnosis (Shirazi et al., 2013). Khazaee‐Pool et al. (2014) also describe the religious and cultural influences of a Muslim woman concerning femininity and modesty as being barriers to mammography screening. Culturally, women are groomed to preserve their breast for their male spouse's satisfaction and this impression precludes attendance to screening clinics that are run by male doctors, thus resulting in low screening rates. Religiously, Islam requires modesty of women, a concept called “*awra*” that results in full body clothing and use of veil with the exception of face and hands when women are in the company of male non‐family members or non‐Muslim men or women. (Boulanouar, 2006). Hence, a good understanding of Islamic beliefs is advocated to aid the delivery of a culturally competent screening that should involve the use of female chaperones who could be female medical staff or female family members (Rassool, 2015).

### 1.2 Study aims

In spite of the plethora of studies on breast cancer screening behaviours emanating from the Middle East and Iran, some of which have examined compliance and adherence (Khodayarian et al., 2016; Samah & Ahmadian, 2012) as well as risk perception, benefits of and obstacles to mammography screening method (Allahverdipour et al., 2011), other factors that can predict the likelihood of the Iranian woman attempting mammography such as awareness, beliefs and fears are not yet fully understood among women in the Tabriz region of Iran. This is one of the few studies that has examined all the possible variables affecting mammography screening practice, according to the mentioned conceptual framework. Therefore, we aimed to identify the pattern of practice of mammography among a sample of Tabriz Iranian women 40 years and older as well as the effects of beliefs, fear and awareness among this subset of women.

## 2 METHODS

### 2.1 Design

This descriptive–correlational study was conducted at Tabriz, East Azarbaijan Province, northwest of Iran from February–July 2017.

### 2.2 Participants and setting

Women who were referred to health centres for health services that included physician appointments, family planning and vaccination were recruited as the study population. At present, Tabriz Health Center has 20 units of urban health center, three units of urban‐rural health center and four units of disease control centre. All centres did not have the same social demographic level but it seemed that the women who were referred to each centre had similarities in terms of socio‐economic variables and in these circumstances, cluster sampling gave us a more representative sample.

In current study, 12 centres were selected for sampling. At first, six out of 10 districts in Tabriz city were chosen randomly along with a random selection of two health centres in each selected district. Inclusion criteria were 40 years and older, of the Iranian origin, resident of Tabriz and no history of breast cancer. A sample size of 140 patients was determined based on a pilot study on 30 women (the percentage of mammography attempts in the sample = %22, α = 0.05, confidence level = 0.95 and error = 0.06). Also considering 10 selected predictor variables to run a multiple regression, this study needed a minimum sample size of 135 patients to achieve 95% power and an effect size equal 0.10 and α = 0.05. Considering a 10% attrition rate, a total of 160 eligible women were selected, of which 152 patients (95% response rate) finally completed the questionnaires.

### 2.3 Ethical considerations

The regional research ethics committee at Tabriz University of Medical Sciences approved this study (IR.TBZMED.REC.1396.250). After obtaining the necessary approvals, women who met the inclusion criteria were identified at selected centres and had the study explained to them as well as the rights of participants. This was followed by obtaining written informed consent.

### 2.4 Data collection

After due permissions were obtained, the study questionnaires were first translated into the predominant local dialects of Persian and Azeri before they were validated for use by 10 Iranian women who eventually were not included in the main study (The Powe Fatalism Inventory was translated first from the English language to the Persian and then from the Persian to the Azeri; translation of other data collection tools followed the same method). Adaptation of the scales was carried out in the series of stages, comprising translation, back‐translation, evaluation by a judging committee including five bilingual faculty members with experience in teaching and researching on the breast cancer and piloting of the pre‐final version in a group of (*N* = 10) women over 40 years old. We examined the face validity and the internal consistency of all the data collection tools with these 10 women participating in the pilot study. Subsequently, participants who were literate completed self‐administered questionnaires while participants who were not literate had the questionnaires administered to them be one of the researchers. Because the research involved the use of five scales with multiple items, completing them all took a minimum of 30 min per patient. Hence, questionnaires were completed during the waiting time for each woman. The following instruments were employed to collect data.

#### 2.4.1 Sociodemographic form

Information collected using this form included age, marital status, religion, literacy, occupation, income, spouse's job and literacy, BMI, pregnancy number and mammogram records within the last 24 months.

#### 2.4.2 Champion's Breast cancer fear scale

This scale designed in 2004 (Champion et al., 2004) contains an eight‐item scale with all items scored on a Likert scale ranging from 1 (strongly disagree)–5 (strongly agree). The scores range between 8–40, with higher scores indicate increasing fear of cancers. The scale has a good internal consistency as indicated by a Cronbach's alpha coefficient of 0.75.

#### 2.4.3 Champion's health belief model scale for mammography screening (CHBMS‐MS) scale

In 1984, Champion (Champion, 1984) developed the Champion's Health Belief Model Scales (CHBMS) based on health belief model constructs (Janz & Becker, 1984; Maiman et al., 1977) to describe health beliefs and breast cancer screening activities. The scale has six subscales of perceived susceptibility, perceived seriousness, perceived benefits, perceived barriers, self‐efficacy and motivation for measuring breast cancer‐related behaviours. Champion (1984) suggested screening through mammography to be a useful activity. In 1999, he revised the CHBMS and developed the Champion's Health Belief Model Scale for Mammography Screening (CHBMS‐MS) (Champion, 1999). This study applied the Farsi version of the CHBMS, which was psychoanalysed by Taymoori and Berry (2009) in Iran and contains eight subscales with 57 items: Susceptibility (3 items), Seriousness (7 items), Benefits BSE (6 items), Barriers BSE (9 items), self‐efficacy (10 items), Health Motivation (7 items), Mammography benefits(6 items) and Mammography barriers(9 items). For this research, six (42 items) out of eight subscales of Farsi version of the CHBMS were used and subscales of Benefits BSE and Barriers BSE were not completed by study participants because they were not relevant to study aim. The scores (six subscales) range from 42–210. All items were scored on a Likert scale ranging from 1 (strongly disagree)–5 (strongly agree). The scale had a Cronbach's alpha coefficient of 0.91 while the subscales have values ranging between 0.79–0.98.

#### 2.4.4 Breast cancer awareness scale

This scale (Asgharnia et al., 2013) for measuring women's awareness of breast cancer was used and is a 16‐item scale with “yes,” “no” and “I do not know” answers where the highest and lowest scores of awareness were 16 and zero, respectively. The internal consistency reliability of this scale is good as evidenced by a Cronbach's alpha coefficient of 0.87.

#### 2.4.5 Powe fatalism inventory (PFI)

This inventory measures cancer fatalistic beliefs. Fatalism is the perception that all events or actions in an individual's life are controlled by an external force (Kobayashi & Smith, 2016). The fatalistic beliefs were measured using a 15‐item scale (Powe, 1995) with “I agree” and “I disagree” answers scored one and zero, respectively. Fatalism scores were determined by adding one point for each “yes” response. Higher scores on the PFI indicate higher levels of fatalism. This scale has a Cronbach's alpha of 0.79.

### 2.5 Data analysis

We excluded participants with missing data (*N* = 8; 5%) and performed a complete‐case analysis. Data were analysed with SPSS version 16. Descriptive statistics including percentage, mean and standard deviation were used to describe the beliefs, fear and awareness about breast cancer. The effects of these variables on mammography were determined by logistic regression analysis. The logistic regression is a mathematical process that produces results that can be interpreted as an odds ratio which is controlled for multiple confounders and its value has been adjusted for the other covariates (Pourhoseingholi et al., 2012). In the current study, it was used to control for numerous confounders (such as age, job and educational level) that based on the literature review, appeared they could influence the behaviour of breast cancer screening with the mammography.

In the logistic regression analysis, participants who adhered to breast cancer screening guidelines (mammogram practice within last 24 month = Yes) were compared with those who did not (mammogram practice within last 24 month = No). A significance level lower than 0.05 was used for statistical tests.

## 3 RESULTS

Most participants (85.3%) were married, partly with an academic degree (30.5%) while a good number of them were housewives (57%). The mean scores for age, number of children and Body Mass Index (BMI) in the participants were 51.51 (*SD=* 8.34), 2.08 (*SD=* 1.28) and 27.70 (*SD=* 4.08) respectively (Table 1). Among participants, only 38.2% had a mammogram record within the last 24 months of which twenty‐one participants (34%) expressed a history of family cancer, twenty‐three individuals (37%) found a suspicious mass on breast self‐examination (BSE), seven women (11%) performed mammography following Clinical Breast Examination (CBE) and subsequent physician's advice. In addition, five persons (9%) were aware of the importance of mammograms in early detection of breast cancer through a personal study, three women (5%) were encouraged by friends and one woman (2%) was influenced by radio‐television advertorials on breast cancer. The participants mentioned the most important barriers to mammography to be lack of time (33.62%), fear of mass discovery (19.83%), high cost (18.97%), unfamiliarity with mammography centres (18.10%) and painfulness of mammography (9.48%).

**Table 1:** Characteristics of study participants

Characteristics	*N* (%)
Marriage status	
Married	128 (85.4)
Single	14 (9.3)
Divorced	8 (5.3)
Education	
Illiterate	24 (15.9)
Under the diploma	37 (24.5)
Diploma	44 (29.1)
Academic	46 (30.5)
Husband education	
Illiterate	29 (19.2)
Under diploma	27 (17.9)
Diploma	38 (25.2)
Academic	38 (25.2)
No spouse	19 (12.6)
Job	
Housekeeper	86 (57.0)
Employee	49 (32.5)
Market	4 (2.6)
Retired	10 (6.6)
Student	2 (1.3)
Husband job	
Unemployed	8 (5.3)
Employee	38 (25.2)
Market	39 (25.8)
Retired	47 (31.1)
No spouse	19 (12.6)
Religion	
Shia	150 (98.7)
Sunni	2 (1.3)
Income status	
Income less than spend	56 (36.8)
Income equal spend	68 (44.7)
Income more than spend	28 (18.4)
Age^a^	51.51 (8.34)
Number of children^a^	2.08 (1.28)
Body Mass Index^a^	27.70 (4.08)

Note

Data presented as *N* (%) unless otherwise indicated.

^a^ Data presented as Mean (*SD*).

With regard to fear, fear of breast cancer averaged 27.26 (*SD* 8.92) of a maximum of 40 points in the participants (Table 2). Overall, 66.4%, 65.8%, 56.6% and 55.3% of participants reported emotions such as fear, discomfort, anxiety and sadness about breast cancer, respectively. Thus, more than half of the study respondents strongly agree that they experience emotions such as fear, anxiousness, anxiety and sadness when thinking about breast cancer.

**Table 2:** Health beliefs and its subscales, fear of the breast cancer and fatalistic beliefs in participants

Health beliefs subscales	Mean (*SD*)	Score range
Susceptibility	2.52 (0.87)	1–4.67
Mammography barriers	2.60 (0.84)	1–4.58
Self‐efficacy	3.11 (0.42)	1–5
Health motivation	3.22 (0.23)	1.63–5
Seriousness	3.31 (0.98)	1–5
Mammography benefits	4.03 (0.77)	1–5
Total (sum of Health beliefs)	131.51 (17.82)	87–189
Sum of fear scores	27.26 (8.92)	8–40
Sum of fatalism scores	5.16 (4.64)	0–15

As shown in Table 2, a mean score of 131.51 (*SD* 17.82) from 210 points was obtained in CHBMS for the participating women. Mean scores of CHBMS sub‐dimensions are as follows: 2.52 (*SD* 0.87) for susceptibility, 2.60 (*SD* 0.84) for mammography barriers, 3.11 (*SD* 0.42) for self‐efficacy, 3.22 (*SD* 0.23) for health motivation, 3.31 (*SD* 0.98) for seriousness and 4.03 (*SD* 0.77) for mammography benefits.

The present study showed that 84.2%, 69.7% and 69.1% of the participants incorrectly answered the questions of awareness on "the relationship between ages of menarche and breast cancer", "painful mass as a symptom of breast cancer" and "nipple discharge as a symptom of breast cancer" respectively. The awareness score averaged 8.74 (*SD* 4.35) from a maximum of 17 points (Table 3).

**Table 3:** Awareness of participants on breast cancer

Items	True response	False response
Painful mass in the breast is one of the symptoms of breast cancer	44 (28.9)	106 (69.7)
Nipple secretion is not a sign of breast cancer	44 (28.9)	105 (69.1)
Lack of symmetry in the breast is one of the symptoms of breast cancer	55 (36.2)	94 (61.8)
Regular exercise is effective in preventing breast cancer	75 (49.3)	75 (49.3)
Vegetables and fruits are effective in preventing breast cancer	99 (65.1)	51 (33.6)
Nipple retraction is one of the most important symptoms of breast cancer	64 (42.1)	85 (55.9)
By increasing the age of marriage, the risk of breast cancer decreases	55 (36.2)	95 (62.5)
The age of the first pregnancy is effective in preventing breast cancer	44 (28.9)	103 (67.8)
By increasing the age menarche, the risk of breast cancer decreases	22 (14.5)	128 (84.2)
Breastfeeding does not play a role in reducing the risk of cancer	85 (55.9)	65 (42.8)
Breast cancer can be hereditary	120 (78.9)	29 (19.1)
Breast cancer usually appears as a painful breast lump	85 (55.9)	64 (42.1)
Early detection of breast cancer develops the result of the treatment	118 (77.6)	32 (21.1)
Breast cancer can be cured if it is diagnosed early	108 (71.1)	42 (27.6)
Breast self‐examination is useful in early detection	119 (78.3)	31 (20.4)
Breast cancer is a common cancer among women	124 (81.6)	25 (16.4)
Breast cancer generally occurs at an advanced age	60 (39.5)	90 (9.2)
Total scores^a^	8.74 (4.35)	

Note

Data presented as *N* (%) unless otherwise indicated.

^a^ Data presented as Mean (*SD*).

The mean fatalism score for the sample was 5.16 (*SD* 4.64) out of a possible maximum score of 15.0 (Table 2) indicating a low degree of fatalism. The number of affirming participants was higher than those who denied the item of "Some people do not want to know if they have a cancer because they do not want to know they may be dying from it". The highest affirming participants with percentages of 82.9%, 77% and 75%, belonged to the items of "people can smoke all their life and if they are not meant to get cancer, they will not get it"; “If someone is meant to have cancer, it does not matter if they eat healthy foods, they will still get cancer" and "If someone gets cancer, a lot of different treatments will not make any difference", respectively.

Using a bivariate logistic regression model with forward approach (maximum likelihood), the variables of demographics, health beliefs, awareness, fear and fatalistic beliefs were included in the model as independent variables (based on literature review age, socio‐economic factors and social level can act as possible confounder and these were adjusted for in the model) and mammogram practice within the last 24 months was considered a dependent variable (a two‐level nominal variable of "yes” for having mammogram practice within the last 24 months" and "no” for not having mammogram practice within the last 24 months"). The results of Omnibus test showed that the error level was <0.01 in the fourth stage, hence, indicating an acceptable fitness of the model. According to Table 4, 37% of variations in the dependent variable can be explained by the logistic model. On the other hand, the Nagelkerke R square determines that 51% of the variability in the dependent variable is explained by the model's independent variables. With the inclusion of four independent variables (susceptibility, health motivation, mammography barriers and self‐efficacy) in the regression model, the amount of explained variance increased to 79.7%. This means that changes in the performing mammography by women can be explained using these four independent variables.

**Table 4:** Predictors of performing mammography in the logistic regression model

Predictors	OR	B	Wald	*p*
Susceptibility	2.83	+1.04	14.30	*p* < .001
Health motivation	2.11	+0.75	5.09	.024
Mammography barriers	0.25	−1.37	16.93	*p* < .001
Self‐efficacy	5.36	+1.68	28.18	*p* < .001
Constant	0.006	−5.10	11.99	.001
Nagelkerke R square	0.51			
Cox & Snell R square	0.37			
−2LL(Log‐ Likelihood)	136.58			

Among the variables affecting the model, self‐efficacy with odds ratios (OR) of 5.36 had the greatest effect on performing mammography. Also, susceptibility (OR = 2.83) and health motivation (OR = 2.11) positively affected the dependent variable. Accordingly, increases in these three variables resulted in rising rates of mammography in the participants. The mammography barriers (OR = 0.25, B = −1.37) have inverse significant effect on the performance of mammography, as with any one score increase in perceived barriers, a 1.37% reduction in performing mammography is likely to occur (Table 4). The effects of other independent variables includes demographic characteristics, fatalism, fear and awareness on the performing mammography were not significant.

## 4 DISCUSSION

The results of this study suggest that attempts to have mammography by women living in Tabriz, Iran, are infrequent as evidenced by just 38.3% of the participants having mammogram records. Talbert (2008) reported a mammography rate of 56% among African‐American women, which is higher than our study. Although their participants showed a significant level of fear and fatalism about breast cancer screening, many individuals seemed receptive in that they expressed concern for their health and further positive personal guidance may perhaps motivate a change towards better screening practices. We posit that this is probably due to the higher education levels of participants and the regular screening programmes organized in their community as reported by that study. According to our findings, the variables of susceptibility, health motivation, mammography barriers and self‐efficacy have significant effects on the mammography screening practice and thus these findings highlight the importance of assessing psychosocial and logistical barriers to screening. In this present study, susceptibility in women was less than the average value and there was a statistically significant relationship between susceptibility and mammography screening practice. Lee‐Lin et al. (2007) found a higher average perceived susceptibility score than ours among Chinese immigrant women in the United States. This finding in the latter study can be attributed to higher knowledge among study participants as well as the systematic screening programmes organized in the United States. Our study findings did not reveal any statistically significant relationship between seriousness and mammography screening practice among the participants. A study by Abbaszadeh et al. (2007) in Kerman showed that mean scores of seriousness, mammography benefits, health motivation and self‐efficacy were higher in women who performed mammography, which is consistent with the current study concerning the effects of other variables on the mammography screening practice, but with the exception of mammography benefits.

We found that self‐efficacy had a significant positive impact on the mammography screening practice, implying improvements in self‐efficacy resulted in increased participation in the mammography. Melvin et al. (2016) have a similar view which found that women who were not confident in their ability to obtain the breast cancer screening had about twice the odds of being out‐of‐date for screening compared with those with greater levels of confidence. Results obtained from the use of data from the Health Information National Trends Survey (HINTS) that asked women how confident they were in terms of obtaining the breast cancer screening also revealed increases in the self‐efficacy of respondents which was promoted by patient‐centred communication (Finney Rutten et al., 2016). Also, the mammography barriers have an inverse significant effect on the mammography screening practices. Abraido‐Lanza et al. (2015) found the strongest predictor of decreased screening to be perceived barriers. The study of Shirzadi et al. (2020) showed that different perceived barriers within various levels (individual, intrapersonal, health systems and community) play influential roles in women's decisions to participate in breast cancer screening programme, which indicates the cultural aspect of perceived barriers in different communities and countries. The findings of the study by Khodayarian et al. (2016) also showed that psychological barriers and maladaptive coping modes including "religious faith," "fatalism" and "avoidance and denial" may be decreased by incorporating religious and cultural belief systems into mammography adherence educational programmes.

The awareness levels of the participants were higher than average value (51%), but there was no statistically significant relationship between awareness and the practice of mammography screening. This finding correlates with another study (Parsa et al., 2008) which observed that the Malaysian women's knowledge was not a significant predictor for the mammography. But in another study (Ghaem et al., 2008), female students in Shiraz were less knowledgeable than the average, which is at variance to what this present study has found. This may be accounted for by the lower average age of the participants in the Ghaem study.

According to the findings of this study, belief in fate and the fear of breast cancer did not have significant effects on the mammography screening practice in this cohort of women. This seems to align with findings by Abraido‐Lanza et al. (2015), where fatalistic beliefs in the Latin American women were not associated with the mammogram screening practices. On the contrary, Charkazi et al. (2013) noticed a significant relationship between fatalistic beliefs and the mammography screening practice in the Iranian Turkmen women, so that women with a high fatalistic belief presented low mammography screening behaviours, an observation associated with low knowledge and low perceived susceptibility. Also, in the study by Andreeva and Pokhrel (2013) concerning immigrant women in Eastern Europe, psychosocial barriers such as attitudes, cultural beliefs and communication problems played an important role in reducing the incentive for cancer screening. The previous studies have shown that culturally based preferences and behaviours related to religion and spirituality have a positive association with screening and other preventive health care (Holt et al., 2005; Wilkinson et al., 2008). However, the culture, language and religious beliefs of this fraction of the Iranian population are different from those of many other parts of the Middle East. There is need for the oncology healthcare providers to understand that there is a very compelling association between the Muslim female patient and her Islamic faith. Iran is a highly religious society which is largely populated by Shia Muslims and Muslim patients generally perceive sickness, suffering and death to be part of one's existence, with illness serving as a reminder of either a spiritual reward, a personal failure in one's dedication to the Islam or a prompting for one to be vigilant concerning one's health (Rassool, 2015). Thus, the authors of this study posit that these religious perceptions may to some extent, be responsible for the low fatalistic views of breast cancer expressed by study participants. This is contrary to the popular view that associates high religious piety with high fatalism and consequent poor health outcomes in some socio‐cultural communities (Leyva et al., 2014). Though not sought for in this study, the authors believe that these religious perceptions of the Muslims towards illness may provide coping mechanisms for female Muslim cohorts with breast cancer.

How a patient perceives her cancer risk is pertinent to the type and amount of information she seeks about it (Valera et al., 2018; Wigfall & Friedman, 2016). Although not sought for in this study, there is an established negative relationship between fatalistic beliefs and early cancer presentation (Beeken et al., 2011). Kobayashi and Smith (2016) recommend an increase in health literacy of the people, to increase public interest in cancer‐related information thus, doing away with perceptions like, "there's not much you can do...." Tang et al. (2018) suggests that tasks perceived to be important can be psychologically or emotionally straining, increasing stress and negative affect such that belief in fate may increase and yield notable effects on subsequent effort. However, the role of fatalism in health behaviour continues to be controversial in literature (Perfetti, 2018), as some researchers have demonstrated an inverse correlation between fatalistic beliefs and healthy behaviours such as cancer screening (Altintas et al., 2017; Crosby & Collins, 2017), while others nullify this hypothesis in other studies (Abraido‐Lanza et al., 2015; Leyva et al., 2014), such as this present study. From this study, one can infer that not all Tabriz Iranian women are fatalistic about cancer. All these findings suggest the complexity of the human health behaviours which requires further studies in this regard.

### 4.1 Limitations and future directions

This study provides a descriptive understanding of predictors of Tabriz Iranian women's participation in mammography. There are several points to consider in developing similar research on the mammography screening practice among women in the future. First, we used a regional sampling strategy that restricted participants to Tabriz and hence findings cannot be generalized nationally. Then, the study sample was largely uneducated women, introducing a sampling bias that may further restrict generalizability. However, given the effective contribution of mammography to early detection of breast cancer, adding knowledge about psychosocial and cultural factors associated to women's mammography screening practice would help to develop a broader understanding of the breast cancer screening behaviours and community‐based interventions.

## 5 CONCLUSION

The mammography screening practice of the Iranian women in Tabriz is inappropriate and mostly affected by barriers of reduced self‐efficacy, motivation and susceptibility. Fatalism is not associated with reduced mammography uptake in this study, but its low presence may be influenced by the religious beliefs of the all‐Muslim cohort of women recruited for this research. Further studies on the association between fatalism and religious beliefs in Muslim participants using population‐specific tools are encouraged. Appropriate training that promotes health beliefs, health literacy and information on the breast cancer screening will eliminate fear, increase self‐efficacy and encourage mammography uptake. Health policymakers are encouraged to reduce the barriers to access mammography through initiatives such as health promotion programmes in the communities and in places of worship to improve women's tendency to undergo breast cancer screening using mammography.

### 5.1 Implication for practice

Nurses in health centres can counselling about the benefits of detecting breast cancer early through mammography screening to alleviate any fears associated with knowing one's risk status. Health education should emphasize on the importance and gains of early detection using mammography. It should include information addressing concerns of the procedure. The development and evaluation of culturally relevant and religiously competent interventions to improve motivation and self‐efficacy and its impacts on the screening behaviours in women of similar cohorts could inform the development of evidence‐based approaches for community health nursing.

## CONFLICT OF INTEREST

The authors declare no conflict of interest.

## AUTHOR CONTRIBUTIONS

LE, AG, AR, AMA, TCO and AN: Study design. LE: Data collection. AG, AR, AMA and TCO: Data analysis/ Interpret the data. LE, AG, TCO and AN: Manuscript writing.

## Data Availability

Data are available on request due to privacy/ethical restrictions.
